# Nucleated red blood cells as a prognostic indicator in dogs with anemia

**DOI:** 10.3389/fvets.2025.1585168

**Published:** 2025-06-19

**Authors:** F. Hollmann, V. Geisen, K. Hartmann, R. Doerfelt

**Affiliations:** LMU Small Animal Clinic, Centre for Clinical Veterinary Medicine, LMU, Munich, Germany

**Keywords:** reticulocytes, rubricytes, hematocrit, regeneration, canine, bone marrow

## Abstract

**Background:**

In human medicine, nucleated red blood cells (NRBCs) in the peripheral blood have been associated with a poor prognosis and increased mortality in critically ill patients. In critically ill dogs, mortality was also significantly associated with high peripheral NRBC count.

**Objective:**

This study aimed to determine the presence of NRBCs in the peripheral blood of dogs with regenerative and non-regenerative anemia and to evaluate the prognostic relevance of NRBCs in anemic dogs. Furthermore, the correlation between NRBCs and other blood parameters was examined.

**Materials and methods:**

Medical records of 254 anemic dogs hospitalized from November 2013 to June 2020 were retrospectively reviewed. Inclusion criteria were a hematocrit of <30%, a minimum age of 6 months, and the presence of a manual blood smear evaluation. Data were analyzed using Fisher’s exact test, Mann–Whitney U test, Kruskal-Wallis test with Dunn’s post-hoc multiple comparison test, and Spearman correlation. *p*-values <0.05 were considered significant.

**Results:**

One hundred ninety-one of 254 patients had NRBCs in their blood smear. The absolute NRBC count was significantly higher in dogs with regenerative anemia [1,514/μl (92–40,810/μl)] compared to dogs with non-regenerative anemia [220/μl (10–5,260/μl); *p* < 0.001]. NRBCs were more often present in dogs with regenerative anemia (141/167) than in dogs with non-regenerative anemia (35/62; *p* < 0.001). The NRBC concentration was not different between surviving and non-surviving dogs (*p* = 0.080). An increase or decrease of NRBCs during hospitalization was also not associated with outcome.

**Conclusion:**

NRBCs commonly appear in the peripheral blood of dogs with regenerative anemia. Their presence and quantity are not associated with survival.

## 1 Introduction

During erythropoiesis in the bone marrow, erythrocyte precursor cells develop through multiple stages until they are released as reticulocytes into the peripheral blood and mature into erythrocytes. The nuclei-containing precursor cells are summarized as nucleated red blood cells (NRBCs). These include rubriblasts, basophilic rubricytes, polychromatophilic rubricytes, and metarubricytes (1–3). The presence of NRBCs in the peripheral blood of newborn puppies is physiological. However, in dogs older than 2 months, these cells can only be found in the bone marrow and are considered pathological when detected in the peripheral blood (4–6).

NRBCs are also named normoblasts or erythroblasts. The pathomechanism leading to the release of these cells into the peripheral blood is not yet fully understood. Increased release of erythropoietin and interleukin (IL)-3 and IL-6 can cause the release of NRBCs (7). Dysregulation of the bone marrow is also a possible cause for the appearance of these immature erythrocytes in the circulating blood (8). In human medicine, the occurrence of NRBCs is associated not only with a variety of diseases, for example, burn injuries, heart failure and multiorgan dysfunction syndrome, but also with increased mortality (2, 8–16).

A few studies in veterinary medicine documented the presence of NRBCs in the peripheral blood of dogs. These precursor cells are frequently seen in dogs with anemia as part of a regenerative response but can also be found in the absence of anemia (17–23). They are reported in dogs with hematopoietic and non-hematopoietic tumors, splenopathy, following splenectomy, in cardiologic diseases, sepsis, and intoxications, as well as in dogs receiving chemotherapy (17–23). Peripheral NRBCs were also found in dogs with heatstroke (24), systemic inflammatory response syndrome (SIRS) (22), acute trauma (25), and generally in critically ill dogs (26). Mortality of critically ill dogs with NRBCs in the peripheral blood was significantly higher compared to NRBC-negative dogs. The highest mortality, with 80% in critically ill dogs, was found in NRBC-positive dogs within the subgroup of those with regenerative anemia (26). In another study, the occurrence of NRBCs in the peripheral blood in dogs with heatstroke was a sensitive and specific marker for mortality and other complications (24). The occurrence of NRBCs was investigated in dogs with various diseases (18). NRBC-positive dogs had significantly lower red blood cell count, hemoglobin concentration, and hematocrit compared to the NRBC-negative control group. Furthermore, NRBCs were more common observed in dogs with severe regenerative anemia (18). However, no current study investigated the prognostic value of NRBCs in anemic dogs, nor the difference of the presence of NRBCs between regenerative and non-regenerative anemia.

This retrospective study aimed to determine the occurrence of NRBCs in the peripheral blood and its prognostic relevance in dogs with regenerative and non-regenerative anemia. Furthermore, this study was intended to clarify correlations between NRBCs and other laboratory values such as hematocrit or reticulocytes.

## 2 Materials and methods

The study protocol was approved by the Ethics Committee of the Centre for Clinical Veterinary Medicine, LMU Munich (AZ 256-21-02-2021).

### 2.1 Case selection

**Figure 1:**
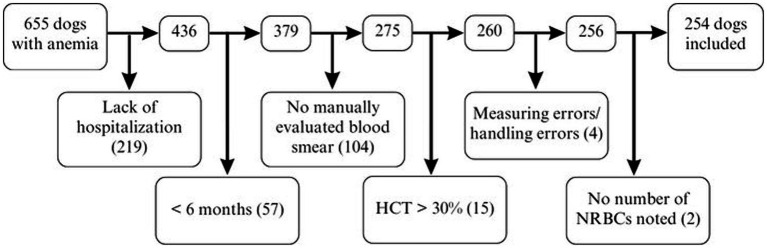
Factors contributing to exclusion in 655 dogs with anemia for NRBC analysis. HCT, hematocrit; NRBCs, nucleated red blood cells.

The electronic medical record system was searched for dogs with anemia between November 2013 and June 2020 at the Clinic of Small Animal Medicine, LMU Munich, Germany. Medical records of dogs older than 6 months that were hospitalized, with a hematocrit of 30% and a manually evaluated blood smear, were included in the study. Medical records of 655 anemic dogs were initially assessed; 401 dogs were subsequently excluded as they did not meet the inclusion criteria (Figure 1). Finally, medical records of 254 dogs were analyzed.

### 2.2 Data collection

Data collection included signalment, case details, complete blood count (CBC), presence of NRBCs, serum parameters, and tests for vector-borne diseases to classify the anemia and evaluate the cause of the anemia.

Anemia was defined as regenerative in dogs with an absolute reticulocyte count of ≥60.000/μl. Manual differential blood count, NRBCs, and blood cell morphology were assessed by examination of modified Wright’s-stained blood smears. Differentiation in the different developmental stages of NRBCs was not performed. Relative NRBC (rNRBCs) count was determined by counting the number of NRBCs per 100 WBCs at 1000 × magnification. If NRBCs were present, WBCs were corrected (cWBC) according to the following formula: cWBC = WBC × [100/(100 + rNRBC)]. Absolute NRBCs (aNRBCs) were calculated by the following formula: aNRBC = cWBC × [rNRBC/(rNRBC + 100)].

Survival was defined as discharge from the hospital with an improving clinical condition.

### 2.3 Statistical analysis

Statistical analysis was performed with a commercial software (GraphPad Prism 5.04; GraphPad Software, San Diego, USA). Normality was analyzed with the D’Agostino-Pearson omnibus test. Data are presented as median (minimal–maximal). The number of NRBC-positive animals was analyzed with the Fisher’s exact test. NRBC values between groups were compared with the Mann–Whitney U test. Among the different causes of anemia, the aNRBC values were analyzed using the Kruskal-Wallis test and Dunn’s *post hoc* multiple comparison test. Correlation was analyzed with Spearman correlation. *p*-values <0.05 were considered significant.

## 3 Results

Male (121 dogs; 47.6%; 46 neutered) and female dogs (133 dogs; 52.4%; 58 spayed) were similarly represented. The median age of all included dogs was 7.0 years (0.5–16.0 years) and the median weight was 15.0 kg (1.0–60.0 kg). The breeds of the included dogs were mixed breed (80), followed by Labrador Retrievers (16), Maltese (8), Pugs (7), Yorkshire Terriers, Cocker Spaniels, Chihuahuas, Bernese Mountain Dogs (6 each), and Golden Retrievers (5). Other breeds were represented with <5 individuals.

**Figure 2:**
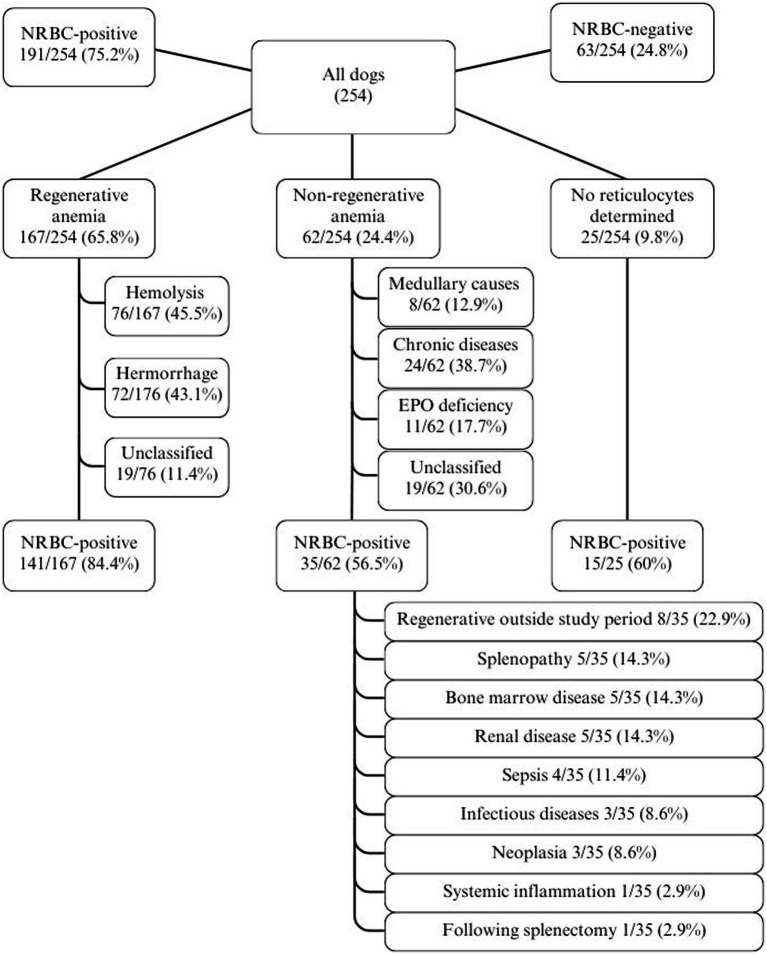
Distribution of cases of anemia in 254 dogs with the proportion/percentage of NRBC-positive dogs. EPO, erythropoietin; non-regenerative, reticulocytes < 60 G/L; NRBC, nucleated red blood cell; regenerative, reticulocytes > 60 G/L.

**Figure 3:**
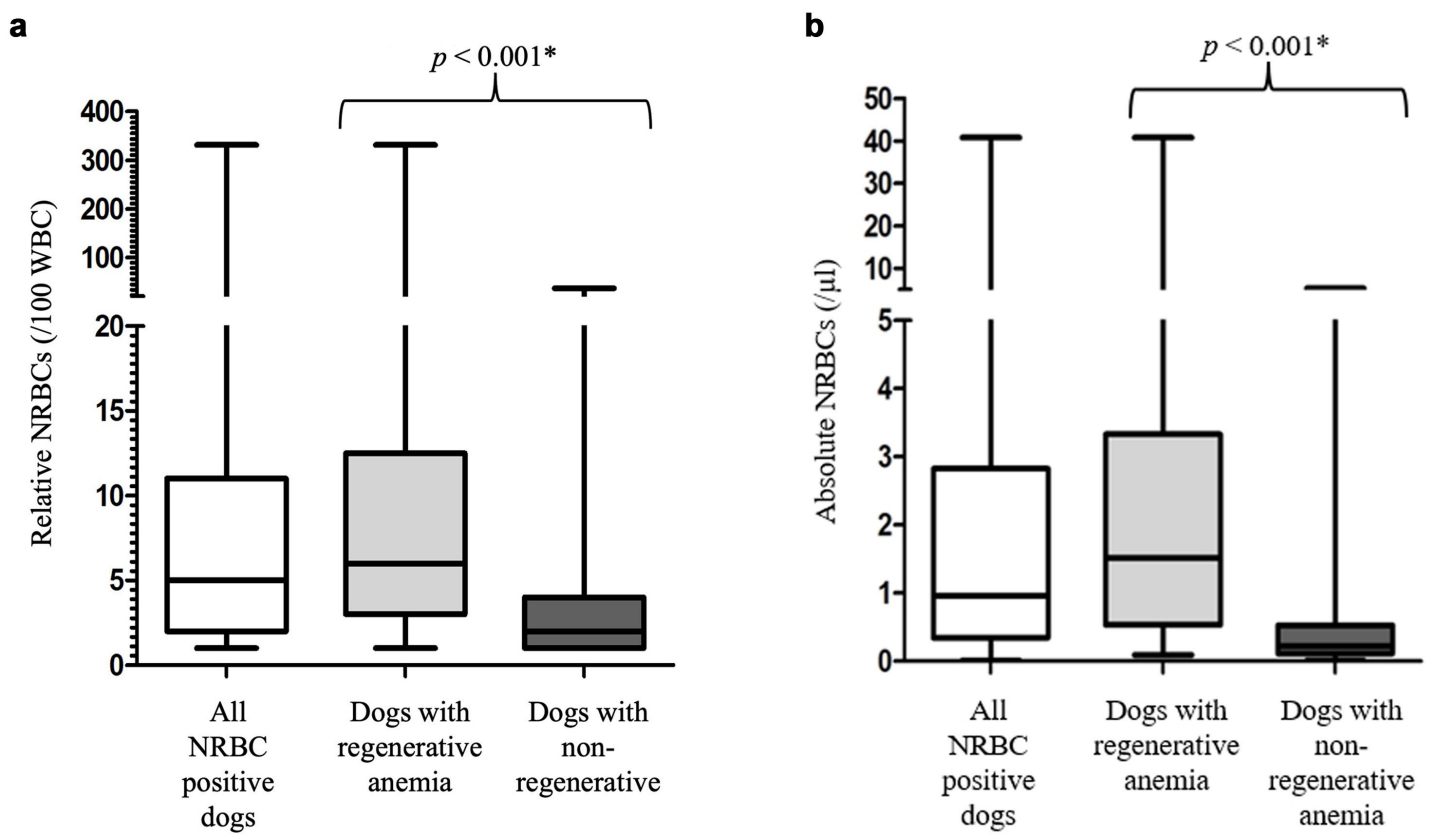
**(a)** Relative number of nucleated red blood cells in the peripheral blood of the 191 NRBC-positive dogs at its first detection. Differences between dogs with regenerative and non-regenerative anemia were analyzed using the Mann–Whitney-U-Test. Non-regenerative, reticulocytes < 60 G/L; rNRBC, relative number of nucleated red blood cells. **(b)** Absolute number of nucleated red blood cells in the peripheral blood of the 191 NRBC-positive dogs at its first detection. Differences between dogs with regenerative and non-regenerative anemia were analyzed using the Mann–Whitney-U-Test. aNRBC, absolute number of nucleated red blood cells; non-regenerative, reticulocytes < 60 G/L; NRBCs, nucleated red blood cells; *p*, significance value (values < 0.05 are considered statistically significant); regenerative, reticulocytes > 60 G/L; WBC, white blood cells; *, significant different *p*-values.

NRBCs were detected in the peripheral blood in 191/254 (75.2%) dogs. The classification into regenerative and non-regenerative and presence of NRBCs is illustrated in Figure 2. Dogs with regenerative anemia were more often NRBC-positive. The amount of rNRBCs and aNRBCs were significantly higher in dogs with regenerative anemia compared to dogs with non-regenerative anemia (Figures 2, 3a,b). Various underlying diseases were identified (Figure 2, Table 1). Of the 62 dogs with non-regenerative anemia, 35 had NRBCs in their peripheral blood (Figure 2).

**Table 1:** Classification of anemia, proportion of NRBC-positive dogs in the number of tested dogs, and median absolute nucleated red blood cell concentration, and range in the peripheral blood in 229 anemic dogs with available reticulocyte count.

Cause of anemia	NRBC-positive (*n*)	aNRBC (/μl)	rNRBC (/100 WBC)
Regenerative			
Hemolysis	67/76	2,196 (92–40,810)^a^	8 (1–331)^b^
Hemorrhage	58/72	1,063 (99–10,760)^c^	5 (1–57)
Unclassified	15/19	1,290 (218–15,300)	5 (1–38)
Non-regenerative			
Medullary disease	5/8	117 (65–520)	3 (1–7)
Chronic disease	13/24	201 (10–5,260)	2 (1–36)
Erythropoietin deficiency	8/11	293 (117–1,177)	2 (1–4)
Unclassified	9/19	266 (87–4,706)	1 (1–10)

aNRBC, absolute number of nucleated red blood cells; *n*, number; non-regenerative, reticulocytes < 60 G/L; NRBC, nucleated red blood cell; rNRBC, relative number of nucleated red blood cells; regenerative, reticulocytes > 60 G/L; WBC, white blood cells.

a Dogs with hemolysis had higher aNRBC compared to all subgroups of non-regenerative anemia (*p* < 0.001) and

b Higher rNRBC compared to the dogs with erythropoietin deficiency (*p* = 0.001; analyzed by Kruskal-Wallis test and Dunn’s post-hoc multiple comparison test).

c Dogs with hemorrhage had higher aNRBC compared to dogs with chronic diseases (*p* < 0.001, analyzed by Kruskal-Wallis test and Dunn’s post-hoc multiple comparison test).

In 167 dogs with regenerative anemia, no difference in aNRBCs between the causes of anemia (hemolysis, hemorrhage, and unclassified regenerative anemia) was observed (*p* > 0.05; Table 1). ANRBC count was not different between subgroups of dogs with non-regenerative anemia (*p* > 0.05). However, concentrations of NRBCs were higher in dogs with hemolysis compared to all subgroups of non-regenerative anemia (*p* < 0.001) and higher in dogs with hemorrhage compared to dogs with chronic diseases (*p* < 0.001; Table 1).

**Figure 4:**
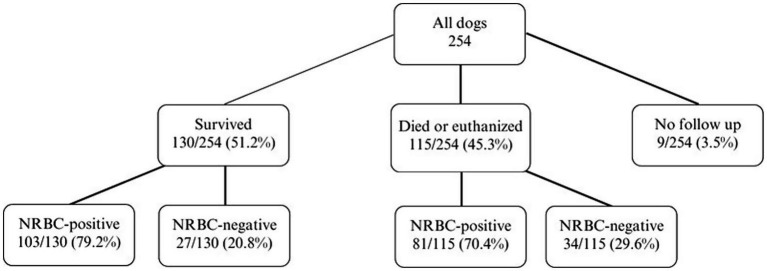
Outcome of 254 dogs with anemia and the number/percentage of NRBC-positive dogs. Differences between survivors and non-survivors were analyzed using the Fisher’s exact test (*p* = 0.386). NRBC, nucleated red blood cell.

**Figure 5:**
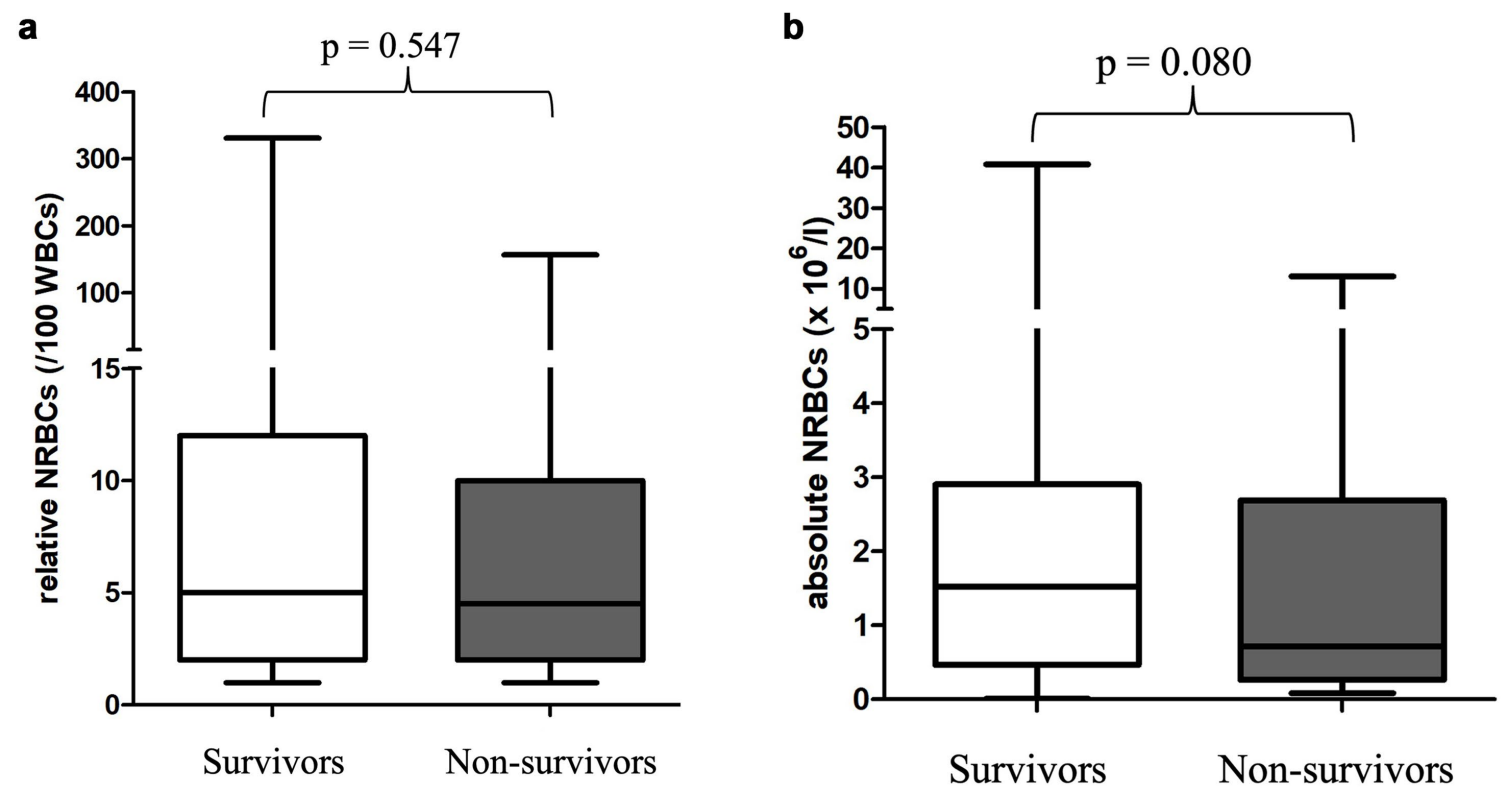
**(a)** Relative nucleated red blood cells in the peripheral blood in 130 surviving and 115 non-surviving anemic dogs at its first detection. Differences between survivors and non-survivors were analyzed using the Mann–Whitney-U-Test. **(b)** Absolute nucleated red blood cells in the peripheral blood in 130 surviving and 115 non-surviving anemic dogs at its first detection. Differences between survivors and non-survivors were analyzed using the Mann–Whitney-U-Test. NRBCs, nucleated red blood cells; *p*-value, significance value (values < 0.05 are considered statistically significant); WBC, white blood cells.

Survival was not associated with the occurrence of NRBCs, nor with absolute or relative NRBC concentration (Figures 4, 5). Neither an increase nor a decrease in aNRBC count during hospitalization was associated with prognosis (Table 2). The survival rate between NRBC-positive and NRBC-negative dogs of the subgroups regenerative (*p* = 0.829) and non-regenerative anemia (*p* = 1.000) was not different. When comparing the surviving and non-surviving dogs, there was no significant difference in the number of NRBCs in either the regenerative anemia group [surviving 1.793/μl (0.099–40.810/μl) vs. non-surviving 1.063/μl (0.092–13.060/μl); *p* = 0.163] or the non-regenerative anemia group [surviving 0.266/μl (0.010–0.724/μl) vs. non-surviving 0.190/μl (0.085–5.260/μl); *p* = 0.539].

**Table 2:** Follow-up values of nucleated red blood cells in the peripheral blood in 103 surviving and 81 non-surviving anemic dogs.

	Survivors		Non-survivors		*p*-value
	*n*	aNRBC (/μl)	*n*	aNRBC (/μl)	
Day 1	103	1,516 (10–40,810)	81	705 (85–13,060)	0.080
Day 2	31	1,157 (119–20,010)	25	1,912 (120–10,000)	0.365
Day 3	31	2,130 (140–32,810)	14	2,540 (160–9,110)	0.722
Day 4	24	1,660 (270–10,490)	12	710 (150–10,350)	0.411
Day 5	18	320 (270–29,230)	8	1,750 (210–8,900)	0.846

Differences between survivors and non-survivors were analyzed using the Mann–Whitney-U-Test. aNRBC, absolute number of nucleated red blood cells; *n*, number of dogs; *p*-value, significance value (values < 0.05 are considered statistically significant).

**Figure 6:**
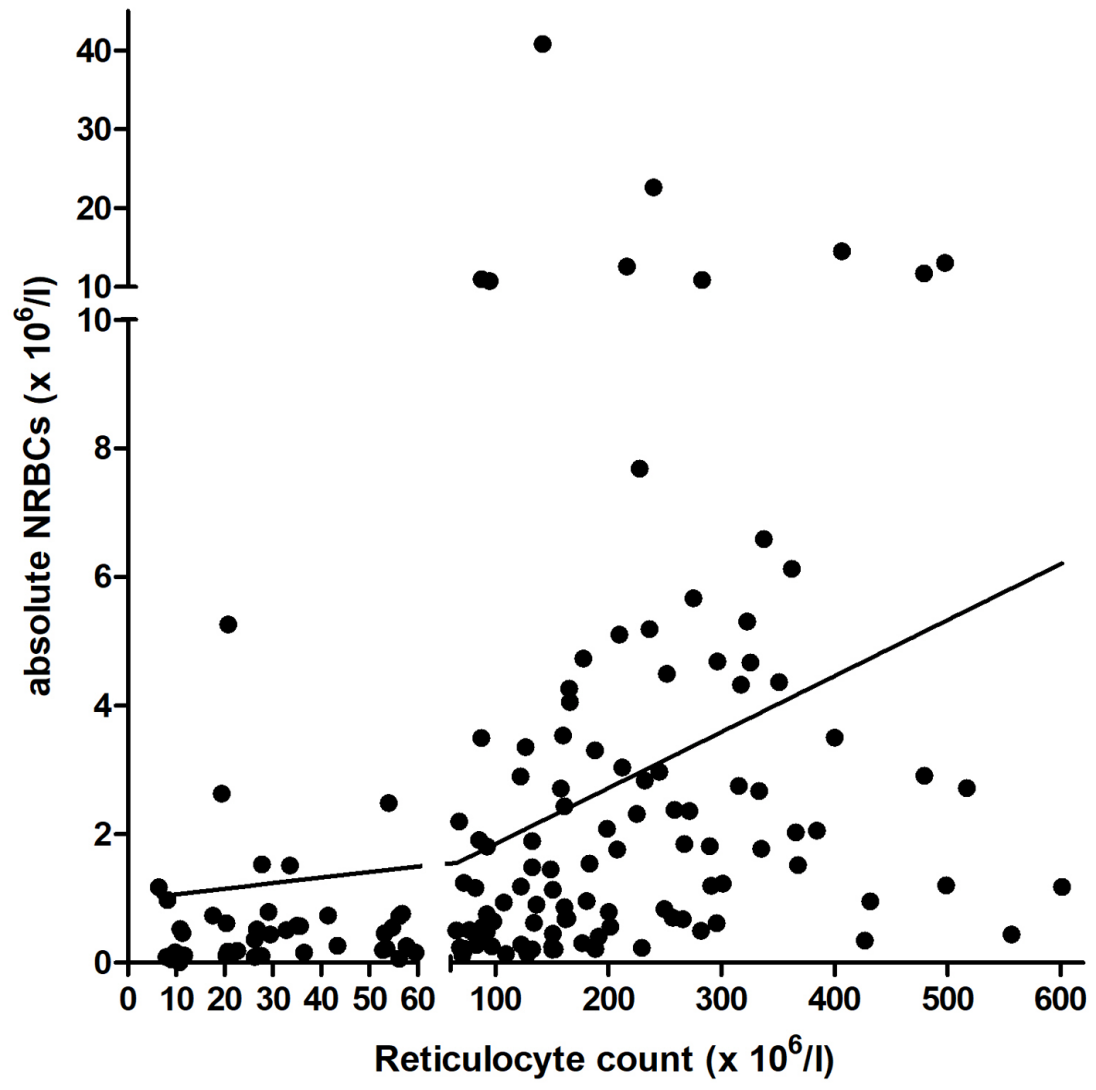
Correlation between the relative and absolute number of NRBCs and reticulocyte count in the peripheral blood in 229 anemic dogs with available reticulocyte count. The y-axis is separated at the cutoff for regenerative anemia (reticulocyte count: 60 × 10^6^/l). NRBCs, nucleated red blood cells.

A mild positive correlation was observed between rNRBC and reticulocyte count and a moderate positive correlation between aNRBC and reticulocyte count was found (Figure 6). In dogs with regenerative anemia, but not in dogs with non-regenerative anemia, the rNRBC and aNRBC concentration correlated mildly with reticulocyte count (Table 3). The correlation between aNRBCs with other hematologic parameters and the bilirubin concentration is presented in Table 4.

**Table 3:** Correlation between the relative and absolute number of NRBCs and reticulocyte count in the peripheral blood in 229 anemic dogs with available reticulocyte count.

	All dogs	Reticulocytes > 60 G/L	Reticulocytes < 60 G/L
rNRBC	*p* = 0.005*, *r* = 0.444*	*p* < 0.001*, *r* = 0.412*	*p* = 0.120, *r* = −0.301
aNRBC	*p* < 0.001*, *r* = 0.555*	*p* < 0.001*, *r* = 0.450*	*p* = 0.810, *r* = −0.048

aNRBC, absolute number of nucleated red blood cells; NRBC, nucleated red blood cell; *p*, significance value (values < 0.05 are considered statistically significant); *r*, Spearman correlation coefficient, if the corresponding *p*-value is < 0.05, *r*-values are considered a statistically significant correlation; rNRBC, relative number of nucleated red blood cells (cells per 100 leucocytes); *, significant values.

**Table 4:** Correlation of absolute nucleated red blood cells with hematologic parameters and bilirubin concentration in the peripheral blood in 254 anemic dogs.

	All dogs (*n* = 254)		Dogs with regenerative anemia (*n* = 167)		Dogs with non-regenerative anemia (*n* = 62)	
	*p*	*r*	*p*	*r*	*p*	*r*
RBC	<0.001	−0.303*	<0.001	−0.281*	0.145	0.252
HCT	0.003	−0.213*	0.023	−0.192*	0.170	−0.237
HGB	0.001	−0.280*	0.002	−0.264*	0.280	−0.188
MCV	0.033	0.366*	<0.001	0.326*	0.248	0.200
MCH	0.004	0.208*	0.017	0.202*	0.658	0.078
MCHC	<0.001	−0.246*	0.007	−0.227*	0.950	0.011
Reticulocytes	<0.001	0.555*	<0.001	0.450*	0.810	−0.048
PLT	0.417	0.059	0.528	0.054	0.394	−0.149
cWBC	0.002	0.411*	<0.001	0.306*	<0.001	0.556*
Monocytes	0.004	0.207*	0.078	0.149	0.072	0.308
Lymphocytes	0.032	0.155*	0.177	0.115	0.008	0.444*
Band neutrophils	<0.001	0.407*	<0.001	0.300*	0.011	0.426*
Segmented neutrophils	0.088	0.360	0.001	0.272*	0.001	0.520*
Eosinophils	0.932	−0.006	0.475	−0.062	0.708	0.066
Basophils	0.972	0.003	0.914	−0.010	0.761	−0.053
Bilirubin	0.084	0.167	0.976	0.003	0.301	0.258

aNRBC, absolute number of nucleated red blood cells; cWBC, corrected white blood cells; HCT, hematocrit; HGB, hemoglobin; MCH, mean corpuscular hemoglobin; MCHC, mean corpuscular hemoglobin concentration; MCV, mean corpuscular volume; *n*, number of dogs; non-regenerative, reticulocytes < 60 G/L; *p*, significance value (values < 0.05 are considered statistically significant); PLT, thrombocytes; *r*, Spearman correlation coefficient, if the corresponding *p*-value is < 0.05 *r*-values are considered a statistically significant correlation; RBC, red blood cells; regenerative, reticulocytes > 60 G/L; *, significant *r*-values.

## 4 Discussion

The present study investigated the occurrence of NRBCs in the peripheral blood, its prognostic impact, and correlations between NRBCs and other laboratory parameters in dogs suffering from anemia. While in human medicine, various studies have been performed demonstrating the prognostic value of NRBCs, veterinary literature is still relatively sparse in this regard. An increased mortality rate in NRBC-positive dogs has already been observed in dogs with heat stroke, acute trauma, and critical illness (24–26).

In the present study, NRBCs were detected in the peripheral blood of 191/254 (75.2%) anemic dogs. Past studies have shown that NRBCs often occur in association with regenerative anemia, but also without the presence of anemia in a variety of conditions. The occurrence of NRBCs is associated with several diseases such as neoplasms, autoimmune hemolytic anemia (IMHA), autoimmune or immune-mediated thrombocytopenia (ITP), trauma, hyperadrenocorticism, dermatological, cardiovascular, urologic, hepatic, gastrointestinal, and neurologic diseases (17). In one study, different diseases were associated with NRBCs in dogs (18). Among the neoplastic diseases, hemangiosarcomas, lymphomas, and mast cell tumors were most frequently associated with NRBCs. NRBCs were also detected in dogs with e. g. IMHA, ITP, ehrlichiosis, trauma, kidney disease, and sepsis. The authors assumed that the release of NRBCs into the peripheral blood in hematological diseases might be induced by the impact on the bone marrow or spleen. In dogs with ehrlichiosis, they suggested that the presence of NRBCs might be attributed to severe bleeding during the acute stage, while in the chronic pancytopenic stage of the disease, NRBCs could also occur secondary to bone marrow lesions and/or spleen involvement (18). Other studies detected NRBCs in the peripheral blood of dogs with splenomegaly or following splenectomy (19, 20). Notably, in one study, two-thirds of the dogs had splenic neoplasms. The authors assumed that anemia and the presence of NRBCs and abnormal red cell morphologies were likely secondary to the tumor or tumor-related hemorrhage (19). NRBCs in the peripheral blood were also present in dogs with severe heart failure and mild to moderate degrees of anemia (21). The increase in NRBCs was associated with increased pyruvate kinase, Glucose-6-phosphate dehydrogenase and 2,3 diphosphoglycerate activity. Therefore, the authors hypothesized that the NRBCs caused changes in enzymatic activities (21). NRBCs were also found in dogs with heatstroke (24). One study detected NRBCs in 90% of the examined dogs. Their presence was unrelated to anemia. A high concentration of NRBCs was associated with a higher rate of secondary complication and death. The authors suggested a correlation of the NRBCs concentration with the severity of the thermal bone marrow injury (24). NRBCs appeared commonly in dogs undergoing chemotherapy due to lymphoma, mast cell tumor or carcinoma (23). Moderate (NRBCs >1%) or severe normoblastemia (NRBCs >5%) were found in 30.3% of these dogs with neoplasms. The authors assumed that the occurrence of NRBCs in the peripheral blood was induced by chemotherapy and related to an altered permeability of the blood-bone marrow barrier, as there was no anemia or other clinical conditions (e.g., heatstroke) usually associated with NRBCs (23). In 24% of dogs with SIRS, NRBCs were detected (22). The authors suspected that the inflammation and/or the reduced oxygen supply to tissues damaged the blood-bone marrow barrier, leading to the release of NRBCs in the peripheral blood (22). A different study investigated the occurrence of NRBCs in dogs with acute trauma, detecting NRBCs in 25.6% of cases (25). Their presence was associated with both regenerative anemia and increased mortality. Since neither regenerative anemia, nor absolute reticulocyte concentration significantly influenced survival, the authors interpreted the detection of NRBCs as premature/inappropriate release from fractures, bone marrow, splenic endothelial injury, or both (25). In a study population of critically ill dogs, 41.5% of the dogs were NRBC-positive (26). NRBCs were found in dogs with regenerative and non-regenerative anemia. The incidence of NRBCs in anemic dogs was higher compared to other critically ill dogs without anemia. The authors assumed that the presence of NRBCs might be caused by hypoxic or inflammatory injury. Due to the lack of correlation between NRBCs and WBCs or inflammatory markers, his assumption could not be further confirmed (26). The presence of NRBCs in a wide range of diseases, with and without anemia, indicates a complex and not yet fully understood pathophysiological mechanism. Their association with conditions ranging from neoplastic diseases to autoimmune diseases, trauma, and systemic inflammatory responses underlines the need for further studies to understand the importance of NRBCs in different disease contexts and to better assess their potential role as a marker of disease severity.

In the present study, NRBCs were found significantly more prevalent in dogs with regenerative anemia compared to dogs with non-regenerative anemia. This aligns with existing veterinary literature associating NRBCs with regenerative responses (18, 25, 26). A retrospective case–control study provided deeper insights into the occurrence of NRBCs in dogs (18). Dogs were categorized into two groups: those with diseases of the hematopoietic system and those with non-hematopoietic diseases, both compared to a control group. Anemia and erythroid regeneration markers (such as polychromasia, anisocytosis, and macrocytosis) were observed more frequently in the group with NRBCs, suggesting a physiological response. Moreover, a positive correlation was noted between the severity of anemia and the presence of NRBCs, indicating a potential association between NRBCs and the regenerative process. Reticulocyte counts were not performed (18). Similarly, in another study, NRBCs were observed in the peripheral blood of traumatized dogs (25). NRBCs were significantly associated with the presence of regenerative anemia, with 6/33 (18.2%) of dogs with NRBCs showing signs of regeneration compared to only 4/96 (4.2%) without NRBCs and signs of regeneration (25). In critically ill dogs, NRBCs also appeared more commonly in dogs with regenerative anemia 15/16 (93.8%) compared to non-regenerative types 7/20 (35%) (26). In the present study, NRBCs were frequently present in anemic dogs, especially in regenerative anemia. This suggests that the presence of NRBCs is probably caused by anemia as a stimulus to the bone marrow and the resulting release of these. This interpretation underscores the importance of considering NRBCs within the context of the broader hematological profile in anemic dogs and, in the case of regenerative anemia, should be interpreted as a sign of regeneration rather than a prognostic marker.

NRBCs were also detected in the peripheral blood of 35 dogs with non-regenerative anemia. All causes of anemia identified in the present study have been previously described in both the veterinary (17–20, 22, 24, 26, 27) and human (2, 28–32) literature as a possible cause for the occurrence of NRBCs in peripheral blood. In 5 dogs, no cause for anemia was apparent. However, a renal disease was present in all of them. The pathophysiology is not yet fully understood, but erythropoietin regulates both, the number of erythroid colony-forming cells (CFU-E), the early release of progenitor cells and influences further differentiation (33). Furthermore, the pathogenesis of anemia due to chronic kidney disease is a multifactorial process. Decreased erythropoietin production, thrombocytopathy, impaired nutrient intake due to hyporexia/anorexia, shortened red cell lifespan, and gastrointestinal bleeding due to uremic gastroenteritis are reported to be among the causes of anemia (34–40). Patients often develop a non-regenerative, normocytic, normochromic, hypoproliferative anemia that is progressive, especially in the end-stage of the disease (34, 35).

The present study did not reveal any differences in the occurrence and concentration of NRBCs between surviving and non-surviving groups. This contrasts significantly with the findings of the study of Dank and others, who reported a markedly higher mortality rate in dogs with NRBCs compared to the control group (24.4% vs. 14.6%) (18). Furthermore, the study demonstrated an increasing mortality rate (*p* < 0.001) with the rise in the absolute number of NRBCs (18). Similar results were observed in previous studies, where the presence of NRBCs was observed at presentation in 95% of dogs with heatstroke (24). Non-survivors exhibited significantly elevated levels of rNRBC and aNRBC, suggesting a correlation between NRBC presence and the severity of thermal injury to the bone marrow. Thus, NRBCs could be used as a prognostic marker in dogs with thermal injury (24). Another study observed a significant association between the occurrence of NRBCs and regenerative anemia in dogs with acute trauma, but neither the presence of regenerative anemia nor the absolute reticulocyte concentration differed significantly between the survival groups (25). This led to the interpretation that the increased mortality associated with NRBCs was attributed to premature/inappropriate release from fractures, bone marrow, or splenic endothelial damage, or both, and not a response to anemia. NRBCs were more prevalent among non-surviving dogs [9/20 (45%) vs. 24/109 (22%)], signifying a probable association between the presence of NRBCs and the extent of injury. Therefore, NRBCs could serve as an additional parameter in trauma scoring systems (25). The mortality in critically ill dogs was also significantly higher in NRBC-positive (54.8%) compared to NRBC-negative dogs (30.5%). However, this difference became statistically insignificant upon excluding anemic individuals (30.0% vs. 28.9%). This discrepancy led the authors to the conclusion that NRBCs might not serve as a reliable prognostic marker in critically ill dogs (26). Since in the present study no difference in the occurrence and number of NRBCs was detected, it is reasonable to assume that NRBCs have no prognostic significance in dogs with anemia but can rather be regarded as a symptom of regeneration. However, the results of the comparative studies suggest a possible prognostic value of NRBCs in certain clinical contexts, which requires further studies.

In the regenerative anemia group of the present study, correlations between aNRBCs and other blood parameters were observed. A mild positive correlation was identified with reticulocyte count, cWBC, and band-, as well as segmented neutrophils. In contrast, Mueller and others also investigated correlations between NRBCs and blood components in critically ill dogs but found no significant associations with any of the blood parameters (26). In dogs with heatstroke, Aroch and co-authors revealed a mild positive correlation between rNRBC and WBC (*r* = 0.36), whereas the correlation between aNRBC and WBC was stronger (*r* = 0.62) (24). The authors assumed that changes in the WBC count might influence the release of NRBCs from the bone marrow, particularly in the context of thermal injury. Another study found that dogs with NRBCs in the peripheral blood showed higher WBC counts, including increased neutrophil counts and a left shift compared to control groups (18). The authors explained the results with the involvement of IL-6, IL-11 and GM-CSF in the regulation of erythropoiesis and their elevation in certain inflammatory conditions. In human medicine, the presence of NRBCs is a marker of severe illness and has been linked to elevated levels of inflammatory markers and cytokines, such as C-reactive protein (CRP) and IL-3, IL-6 and IL-8, which are known to stimulate neutrophil production and release (7, 41). Conditions that result in the appearance of NRBCs in peripheral blood are also associated with increased neutrophil counts, reflecting the body’s response to severe stress and inflammation (7). In dogs with primary immune-mediated hemolytic anemia (IMHA), similar results were observed (42). These dogs exhibited significantly higher concentrations of CRP, IL-2, IL-10, keratinocyte chemoattractant protein, and monocyte chemoattractant protein-1 compared to healthy controls. The correlation between aNRBC and WBC, as well as neutrophil granulocytes in the present study, can also be interpreted as a sign of regenerative response. Prolonged hemolysis, for example, triggered by strong stimuli to the bone marrow, can lead to increased leukopoiesis and thrombopoiesis, which could manifest in altered blood parameters (28). Overall, the findings underscore that the presence of NRBCs in peripheral blood is commonly accompanied by leukocytosis, particularly neutrophilia. This response likely represents a physiological reaction to underlying pathological conditions, such as significant inflammation or systemic stress. These findings also show the complex interaction between NRBCs and hematological parameters, which underlines the need for further studies.

Due to its retrospective nature, this study has limitations. A complete diagnostic work-up was not available for all dogs and the assessment of NRBCs in manual blood smears was not conducted every day. The heterogeneous patient population in terms of underlying diseases presents an additional limitation. Future studies focusing on a specific cause of anemia could provide more precise insights into the prognostic significance of NRBCs.

In conclusion, NRBCs are prevalent in anemic dogs, particularly in cases of regenerative anemia, yet they have no prognostic relevance in these patients, rather they should be considered as a symptom of regeneration. Further prospective studies focusing on specific diseases are needed to elucidate the precise role of NRBCs in different pathological conditions.

## Data Availability

The raw data supporting the conclusions of this article will be made available by the authors, without undue reservation.
